# Chloroplast Genome Analysis for Genetic Information and Authentication in Five *Barleria* Species

**DOI:** 10.3390/genes13101705

**Published:** 2022-09-22

**Authors:** Sanit Kaewdaungdee, Runglawan Sudmoon, Tawatchai Tanee, Shiou Yih Lee, Arunrat Chaveerach

**Affiliations:** 1 Department of Biology, Faculty of Science, Khon Kaen University, Khon Kaen 40002, Thailand; 2 Faculty of Law, Khon Kaen University, Khon Kaen 40002, Thailand; 3 Faculty of Environment and Resource Studies, Mahasarakham University, Maha Sarakham 44150, Thailand; 4 Faculty of Health and Life Sciences, INTI International University, Nilai 71800, Negeri Sembilan, Malaysia

**Keywords:** *Barleria* species, chloroplast genome, phylogenetic relationships

## Abstract

In order to authenticate the genomic information of *Barleria* *cristata* L., *B. lupulina* Lindl., *B. repens* Nees, *B. siamensis* Craib, and *B. strigosa* Willd, cp genomes were investigated. They revealed a general structure with a total size of 151,997–152,324 bp. The genomes encoded a total of 131 genes, including 86 CDS, 37 tRNA, and 8 rRNA genes. Other details found were as follows: different numbers and types of SSRs; identical gene content, which is adjacent to the border regions, except for *B. strigosa*, that revealed a shorter *ndh*F gene sequence and lacked the *ycf*1 gene; slightly different genetic distance values, which can be used for species identification; three distinct gaps of nucleotide variations between the species located at the intergenic spacer regions of the LSC and CDS of the SSC; three effective molecular markers derived from divergent hotspot regions, including the *ccs*A-*ndh*D, *ndh*A-*ndh*H-*rps*15, and *ycf*1. The genetic relationships derived from the cp genome and the CDS phylogenetic trees of *Barleria* and the 13 genera in Acanthaceae and different families, Scrophulariaceae and Phrymaceae, showed similar results. The six *Barleria* species as monophyletic groups with inner and outer outgroups were found to have perfect discrimination. These results have helped to authenticate the five *Barleria* species and the six genera in Acanthaceae.

## 1. Introduction

*Barleria* (Barlerieae) species belong to the Acanthaceae family. They have a large, polymorphic, widespread genus of perennial herbs, shrubs, and rarely climbers, and comprise about 300 species worldwide [1]. They are distributed in Africa and Asia, with the highest diversity in tropical East Africa and southern Africa. *Barleria* can be easily distinguished from other genera of the Acanthaceae by a combination of three characters, including a four-part calyx with two large outer segments and two smaller inner ones, spheroidal pollen grains, and double crystoliths in the epidermal cells [2]. The *Barleria* species are crucial plants in folk traditional medicine and have ornamental potential. Numerous bioactive compounds, such as alkaloids, flavonoids, phenolic compounds, iridoid glycosides, quinones, quercetin, terpenoids, steroids, and tannins [1,3], have been reported from various *Barleria* species, corresponding with medicinal properties such as anti-oxidant, anti-inflammatory, analgesic activity, anti-leukemic, anti-tumor, anti-hyperglycemic, anti-virus, and anti-bacterial activity [4,5,6,7]. The *Barleria* species can be made into natural products, such as medicines, cosmetics, and nutraceutical supplements. These uses, however, correspond only with certain species. Some species have similar morphological characters, especially leaf morphology and flower color [8], which may lead to species misidentification. Several studies reported using a small sequence as a genetic marker, such as *rbc*L, *rps*16, *mat*K, *trn*L-*trn*F, *trn*S-*trn*G, and *ndh*F-*rpl*32-*trn*L, and a nuclear ribosomal internal transcribed sequence (nrITS) to resolve taxonomic problems within the genus [2,8,9,10,11,12,13]. These short sequences used as genetic markers, called DNA barcodes, have rather limited information due to a limited number of nucleotide sequences; thus, there are still some controversies regarding the identification and lineage categorization of the *Barleria* species. 

With the development of high-throughput sequencing technologies, chloroplast DNA (cpDNA) has been widely employed in plant systematics to overcome the species identification problem [14,15]. The chloroplast genome is usually maternally inherited in plants and consists of a quadripartite circular double-stranded DNA molecule, a large single-copy (LSC) region, a small single-copy (SSC) region, and two copies of inverted repeat (IR) regions [16,17,18]. In the higher plant cells, cpDNAs are double-stranded molecules with a relatively tiny size, ranging from 35 to 217 kilobases [19,20,21]. The cpDNAs represent highly conserved genetic information in terms of genome structure, gene content, and order due to single-parent inheritance, being haploid, and its non-recombinant nature [22,23,24]. Therefore, the chloroplast genome plays an indispensable role in the molecular evolution, phylogeny, and molecular markers of plants [25,26,27,28,29]. Hence, in the current study, we sequenced and characterized five *Barleria* chloroplast genomes and compared them to *B. prionitis* and other genera of the Acanthaceae in order to utilize the data for interpreting genetic relationships between species. Furthermore, we wanted to develop potential markers for authenticating *Barleria* species identification accurately, leading to the safe human application of these plants.

## 2. Material and Methods

### 2.1. Plant Materials 

The sampling was carried out from January–April 2021. Young, fresh leaves of five *Barleria* species, including *B. cristata*, *B. lupulina*, *B. repens*, *B. strigosa*, and *B. siamensis*, were collected for total genomic DNA extractions. Aside from *B. repens*, which originated from the Nakhon Ratchasima province, the other four *Barleria* species were from natural populations and ex-situ sites located in the Chiang Mai province, Thailand. All the collections and experiments were performed in accordance with the relevant local guidelines and regulations, and the voucher specimens were kept at the Department of Biology, Faculty of Science, Khon Kaen University, under the collection number A. Chaveerach 1095–1099. The species identification was carried out by the corresponding author and is based on the taxonomic classification proposed by Shendage and Yadav (2010) [30]. Upon collection, the leaf samples were kept in zip-lock bags filled with silica gel and were transported to respective local laboratories for total genomic DNA extractions.

### 2.2. DNA Extraction

The leaf samples were subjected to total genomic DNA extraction using the DNeasy Plant Mini Kit (QIAGEN, Hilden, Germany), based on the manufacturer’s protocol. The integrity of the genomic DNA was detected via gel electrophoresis using a 1% agarose gel. The purity and quantity of the DNA were estimated using the Qubit™ 4 Fluorometer (Thermo Fisher Scientific, Waltham, MA, USA).

### 2.3. Chloroplast DNA Sequencing, Assembly, and Annotation

A 350-bp paired-end library was prepared using the TruSeq DNA Sample Prep Kit (Illumina, San Diego, CA, USA) to obtain 150-bp pair-end reads. Next-generation sequencing (NGS) was conducted on an Illumina NovaSeq platform (Illumina, San Diego, CA, USA). A total of 6 Gb of raw NGS data was obtained and fed into the NOVOPlasty 2.7.2 [31] pipeline for genome assembly. The genome assembly was performed by selecting the *rbc*L gene of *Barleria prionitis* (GenBank accession NC_048478) as the seed sequence. A contig was obtained at the end of the assembly process, and a gene annotation was carried out using GeSeq [32]. The annotated genome was manually checked for errors, and the circular chloroplast genome map was illustrated using OGDRAW v.1.3.1 [33]. The annotated genome sequences were deposited into the National Center for Biotechnology Information (NCBI) GenBank database.

### 2.4. Repeat Analysis

The simple sequence repeats (SSRs) were identified using MISA-web [34]. The parameters for the minimum number of repeats were set at 10, 4, 4, 3, 3, and 3 for mono-, di-, tri-, tetra-, penta-, and hexanucleotides, respectively. For the identification of large repeats, the REPuter program was used to identify forward, palindromic, reverse, and complement repeats [35]. The minimum repeat size was set at 30 bp, and the Hamming distance was set to 3. For tandem repeats, the number of tandem repeats was analyzed using the tandem repeat finder v.4.09 with advanced options including 2, 2, 7 for the alignment parameters, 80 for the minimum alignment score, and 500 for the maximum period size, respectively [36]. 

### 2.5. IR Border Region and Chloroplast Genome Comparison

The boundaries and junctions of the inverted repeat (IR) regions were visualized using IRscope [37] and were edited using Adobe Photoshop CS6 (Adobe, San Jose, CA, USA). The comparative analyses of the six *Barleria* chloroplast genomes were analyzed using mVISTA [38] with Shuffle-LAGAN mode [39]. 

### 2.6. Genetic Distance and Sequence Divergence Snalysis

A multiple sequence alignment was carried out on the six chloroplast genome sequences of *Barleria* using MAFFT v.7 [40,41]. A genetic distance was performed using the MEGA-X with the Kimura 2-parameter model. The rate of variation among the sites was modeled with a γ distribution. All positions containing gaps and missing data were eliminated using the complete deletion option [42,43]. To determine the nucleotide diversity (Pi) in three different regions of the chloroplast genome, including the LSC, SSC, and IR regions, an analysis was performed using DnaSP v.5.10.01 [44]. The window length was set at 1000 bp, and the step size was set at 500 bp. The chloroplast genome sequence of *B. prionitis* was selected as the reference genome.

### 2.7. Phylogenetic Tree Analysis

Phylogenetic trees were reconstructed using two different sequence datasets, including the complete chloroplast genome sequences, a concatenated dataset of the protein-coding sequences (CDSs), and four regions of nucleotide diversity. A total of 18 species from Acanthaceae were included in the analysis as follows: six from the Barlerieae tribe, five from Ruellieae (*Echinacanthus attenuates* (NC_039762), *E. lofouensis* (NC_035876), *E. longipes* (NC_039761), *E. longzhouensis* (NC_039678), and *Strobilanthes cusia* (NC_037485); four from Justicieae (*Clinacanthus nutans* (NC_042162), *Justicia adhatoda* (NC_047476), *J. flava* (NC_044862), and *J. leptostachya* (NC_044668); two from Acantheae (*Aphelandra knappiae* (NC_041424) and *Blepharis ciliaris* (NC_046601); one from Andrographidieae (*Andrographis paniculate* (NC_022451)). Two species, *Scrophularia dendata* (NC_036942) and *Erythranthe lutea* (NC_030212), were included as the outgroups. For the CDS dataset, the CDS sequences for each species were extracted using FeatureExtract-1.2 [45]. All sequence datasets were aligned using MAFFT v.7 [41]. 

The phylogenetic analyses were performed using both the maximum likelihood (ML) and the Bayesian inference (BI) methods. The substitution models with the best fit were estimated using jModelTest 2.1.10 [46,47] based on the Akaike Information Criterion (AIC) [48]. A maximum likelihood analysis was constructed on IQ-Tree v.1.4.2 under the generalized-time-reversible (GTR) model with gramma (+G) and 1000 ultrafast bootstrap replicates [49,50]. A Bayesian inference analysis was performed using MrBayes on XSEDE v.3.2.7a by the Cyberinfrastructure for Phylogenetic Research (CIPRES) Science Gateway v.3.3 [51,52]. A Markov Chain Monte Mont (MCMC) simulations were run for 2 million generations, and sampling trees were run every 100 generations [53]. The resulting trees were visualized and edited using FigTree v.1.4.4 [54]. 

## 3. Results

### 3.1. Barleria Chloroplast Genome Organization and Features

**Figure 1:**
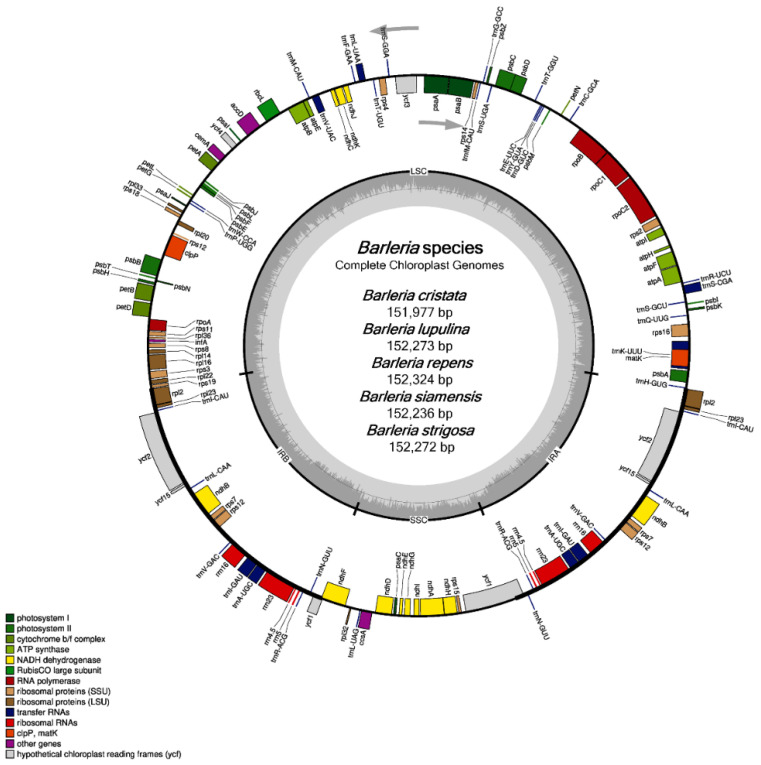
Gene map of the five species of *Barleria* chloroplast genomes. The inside circle genes are transcribed clockwise, whereas the outside circle genes are transcribed counter-clockwise; the color codes are described for the different function groups of the genes; the thick lines indicate the boundary of the inverted repeats (IRA and IRB), demarcated between the large single-copy (LSC) and small single-copy (SSC) regions; the dark gray area in the inner circle represents the genomic GC content, whereas the light gray indicates the AT content, respectively.

All of the five chloroplast genomes revealed a general cp structure that is quadripartite, containing a large single-copy (LSC) region, a small single-copy (SSC) region, and a pair of inverted repeats (IRs) (Figure 1). The genome size of the five *Barleria* species ranged from 151,997 bp (*B. cristata*) to 152,324 bp (*B. repens*). For each species, the LSC regions ranged from 83,650 bp (*B. cristata*) to 83,929 bp (*B. siamensis*) and the SSC regions ranged from 17,677 bp (*B. cristata*) to 17,804 bp (*B. siamensis*), while the IR regions ranged from 25,296 bp (*B. siamensis*) to 25,367 bp (*B. repens*). The chloroplast genomes of the five *Barleria* species were predicted to encode a total of 131 genes, including 86 protein-coding (CDS), 37 tRNA, and 8 rRNA genes. Among them, 18 genes were duplicated in the IR regions, including seven CDS, seven tRNA, and four rRNA (Figure 1 and Table 1). A total of 18 genes that contain introns (s) were detected. The *clp*P and *ycf*3 genes contained two introns, while the other 16 genes, including *atp*F, *ndh*A, *ndh*B, *pet*B, *pet*D, *rrn*23, *rpl*2, *rpl*16, *rpo*C1, *rps*16, *trn*A-UGC, *trn*I-GAU, *trn*K-UUU, *trn*L-UAA, *trn*S-CGA, and *trn*V-UAC, contained one intron (Table 2 and Table 3). The overall GC content was 43.5 % in all of the species, while the GC content of each region was 36.4–36.5%, 32.4–32.7%, and 38.3–38.4% for the LSC, SSC, and IR, respectively (Table 1).

### 3.2. Repeated Sequence Analysis

**Figure 2:**
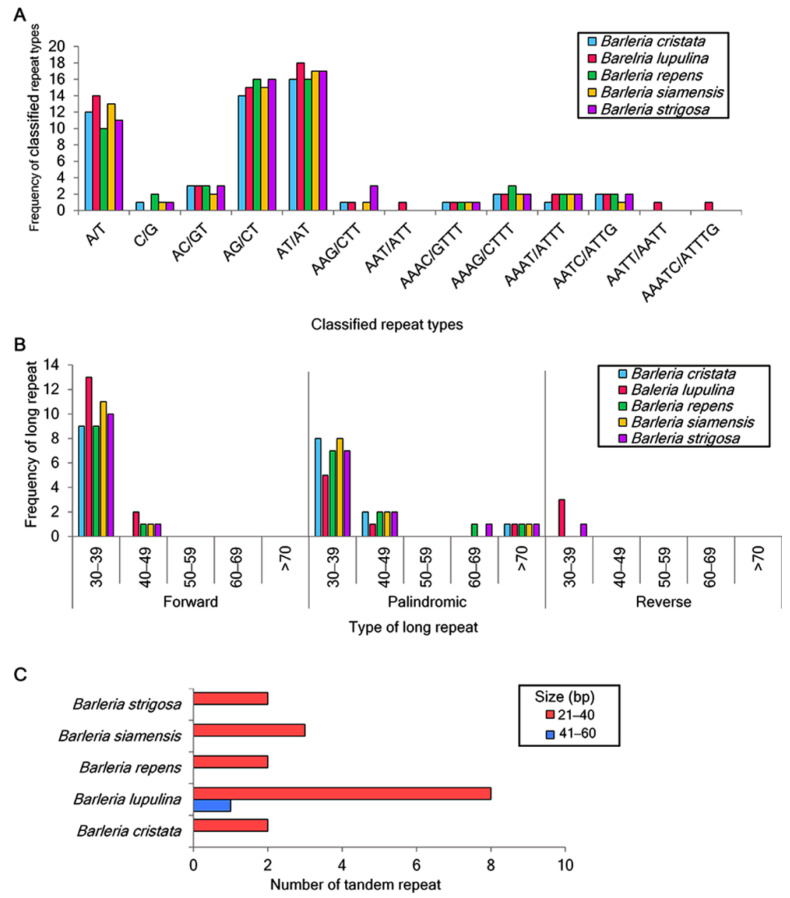
Repeated sequence analysis in the five *Barleria* species. (**A**) Number of classified SSR repeat units; (**B**) distribution and frequency of long repeats, including forward, palindromic, and reverse repeats; (**C**) number of tandem repeats.

A total of 53, 61, 55, 56, and 58 of the SSRs were identified in the chloroplast genome sequences of *B. cristata*, *B. lupulina*, *B. repens*, *B. siamensis*, and *B. strigosa*, respectively. For the mononucleotide repeats, the A/T repeats were found to be more abundant than the C/G repeats. For the dinucleotide repeats, the nucleotide combination of AT/AT was the most abundant, of which 18 were identified in *B. lupulina* and *B. repens*, 17 were identified in *B. siamensis* and *B. strigosa*, and 16 were identified in *B. cristata.* This was followed by the nucleotide combination of AG/CT, of which 16 were identified in *B. repens* and *B. strigosa*, 15 were identified in *B. lupulina* and *B. siamensis*, and 14 were identified in *B. cristata*. Trinucleotide repeats were detected in all species except for *B. repens*. Tetranucleotide repeats were identified in all of the study species, and the repeat AATT/AATT was found only in *B. lupulina***.** Pentanucleotide repeats were only detected in *B. lupulina* (Figure 2A). 

A total of 135 long repeats were also analyzed in the five *Barleria* chloroplast genomes. Most of the long repeats were in a sequence size between 30 and 39 bp, and the reverse long repeats were only detected in *B. lupulina* and *B. strigosa* (Figure 2B). For the tandem repeats, fifteen repeats were detected, including two, nine, two, three, and two in *B. cristata*, *B. lupulina*, *B. repens*, *B. siamensis*, and *B. strigosa*, respectively. The majority of these repeats were between 21 and 40 bp in length. As for the tandem repeat with a length of 41–60 bp, it was only found in *B. lupulina* (Figure 2C). 

### 3.3. Comparison of Border Regions in Barleria Chloroplast Genomes

**Figure 3:**
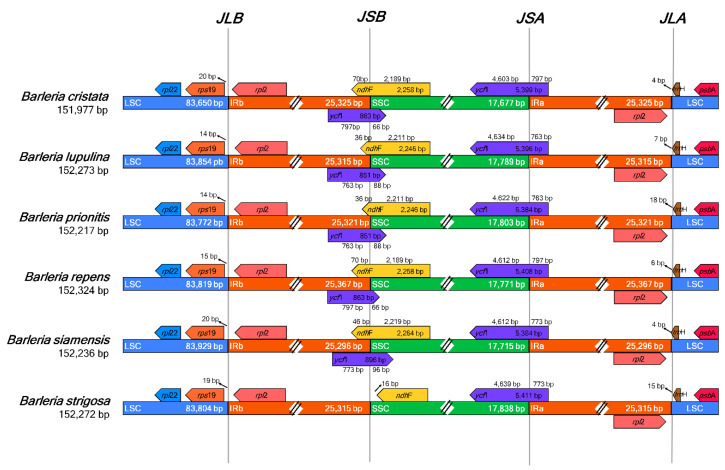
Comparison of the large single-copy (LSC), small single-copy (SSC), and a pair of an inverted repeat (IR) region borders of the six *Barleria* chloroplast genomes. The boxes below and above the lines indicate the adjacent border gene revealing the point differences as a shorter *ndh*F gene sequence and a lack of the *ycf*1 gene in *B. strigosa*.

The six species of *Barleria* revealed identical gene content adjacent to the region borders (Figure 3). The genes *trn*H and *rpl*2 were adjacent to the junction between the LSC and IRA (JLA), placed in the LSC and IRA regions, respectively. At the junction between the LSC and IRB regions (JLB), the genes *rps*19 and *rpl*2 were placed adjacent to the junction, in the LSC and IRB regions, respectively. The *ycf*1 gene was detected crossing the junctions between the SSC and IRA (JSA) for all *Barleria* species; however, the genes *ycf*1 and *ndh*F were both detected crossing the junction between the SSC and IRB (JSB) for all species except for *B. strigosa***,** which revealed the point differences as a shorter *ndh*F gene sequence and a lack of the *ycf*1 gene. 

### 3.4. Genetic Distance Analysis

For the genetic distance analysis, the *Barleria* sequences indicated slightly different genetic distance values to separate the interspecific species, ranging from the lowest value of 0.00241 (between *B. lupulina* and *B. prionitis*) to the highest value of 0.01125 (between *B. prionitis* and *B. strigosa*) (Table 4). Even though there are not many genetic differences, the values can be used for species identification. 

### 3.5. Interspecific Genome Variations and Nucleotide Diversities

**Figure 4:**
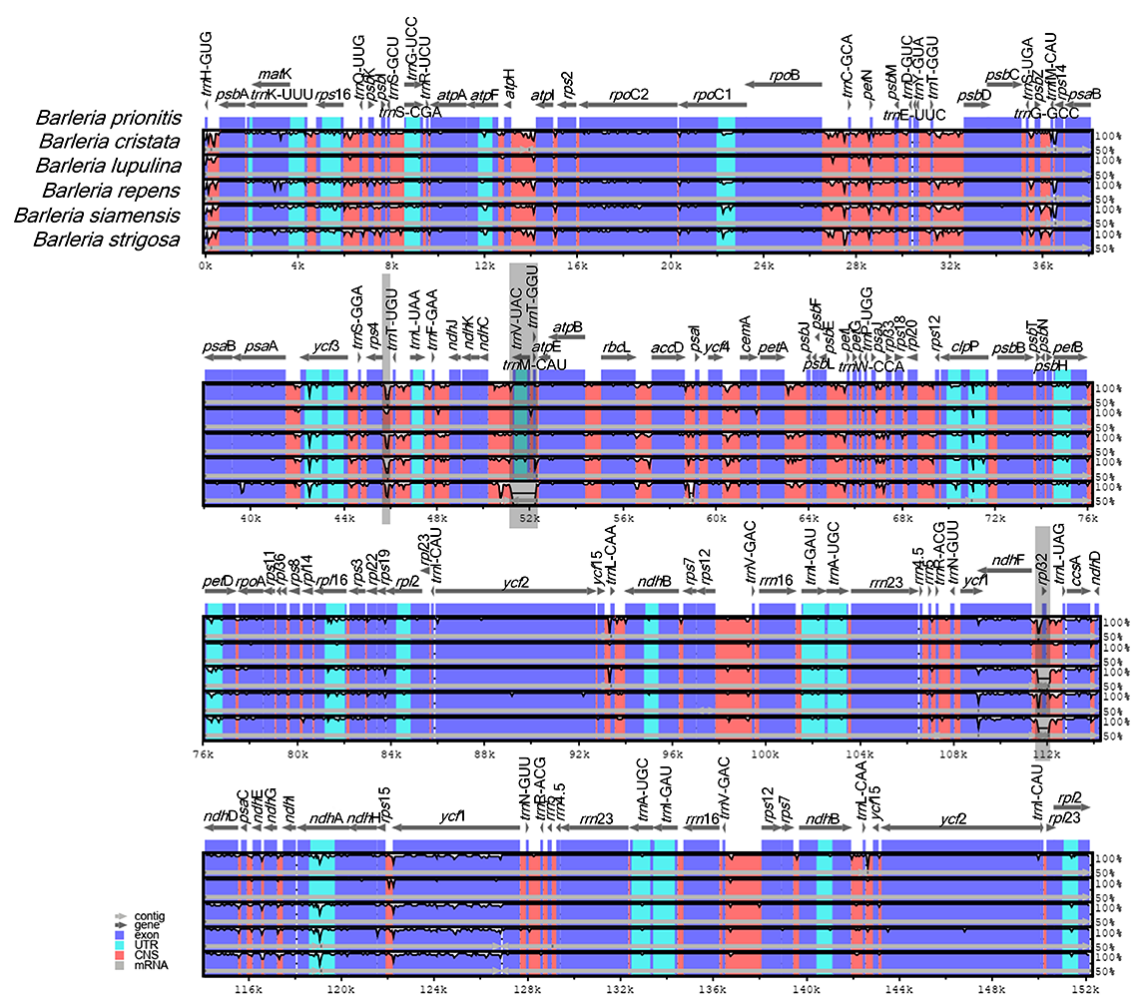
Sequence alignment comparison of the *Barleria* chloroplast genome using mVISTA, with *B. prionitis* as a reference. The purple bars represent exons; the pink bars represent non-coding sequences (CNS); the light-blue bars represent tRNA and rRNA regions; the gray arrows above the aligned sequences indicate the genes and their orientations; the x-axis represents the number of bases in the aligned sequences; the y-axis represents the percent identity from 50–100%; the dark gray bars indicate variation regions in the genomes.

A genome alignment of the six *Barleria* species revealed at least three distinct gaps that represent nucleotide variations among the species (Figure 4, gray area). The variations were in the form of insertion–deletion (InDels). Two of them are located in the intergenic spacer regions (IGSs) *rps*4*-trn*T-UGU and *trn*V-UAC*-trn*T-GGU, both from the LSC region, in which the latter is specifically present in *B. strigosa*. The third variation was located at the CDS *rpl*32 of the SSC region, specifically present in *B. repens* and *B. strigosa*. 

### 3.6. Recognition of Highly Variable Regions within the Barleria Chloroplast Genomes

**Figure 5:**
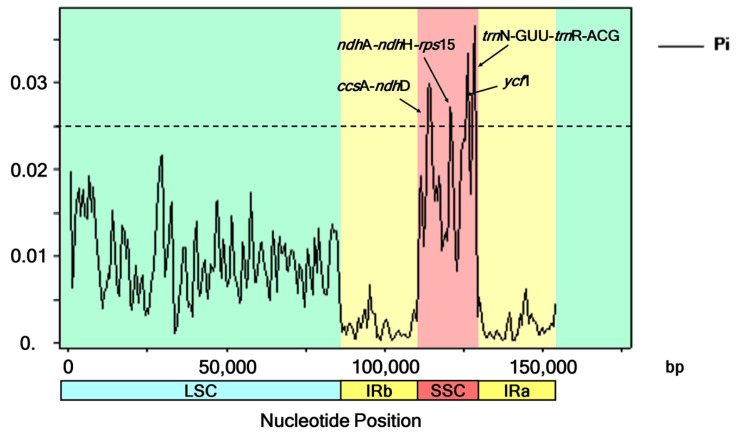
Nucleotide variability values were compared between the six *Barleria* chloroplast sequences using a sliding window analysis (window length is 1000 bp; step size is 500 bp). The x-axis and y-axis indicate the position of the midpoint of the nucleotide diversity of each window.

The six *Barleria* aligned sequences with a total of 154,153 nucleotide sites revealed 2920, 1869, 806, and 121 variable (polymorphic) sites in the complete chloroplast genomes, LSC, SSC, and IR sequences, respectively. Four divergent hotspot regions were identified in all six *Barleria* cp genomes, including three intergenic spacer regions (*ccs*A*-ndh*D, *ndh*A*-ndh*H*-rps*15, and *trn*N-GUU*-trn*R-ACG) and a protein-coding region (*ycf*1). Their nucleotide diversity values (Pi) ranged from 0.0267 to 0.0365. The region of *trn*N-GUU*-trn*R-ACG (1034 bp in length) showed the highest variability with a Pi value of 0.0365. The next two subsequent regions were *ycf*1 (1026 bp, Pi = 0.0334) and *ccs*A*-ndh*D (2150 bp, Pi = 0.0249). The diversity level of *ndh*A*-ndh*H*-rps*15 was the lowest, with 1536 bp and a Pi value of 0.0267. All of the mentioned divergent hotspot regions were detected in the SSC regions (Figure 5). 

### 3.7. Phylogenetic Relationship Analysis

**Figure 6:**
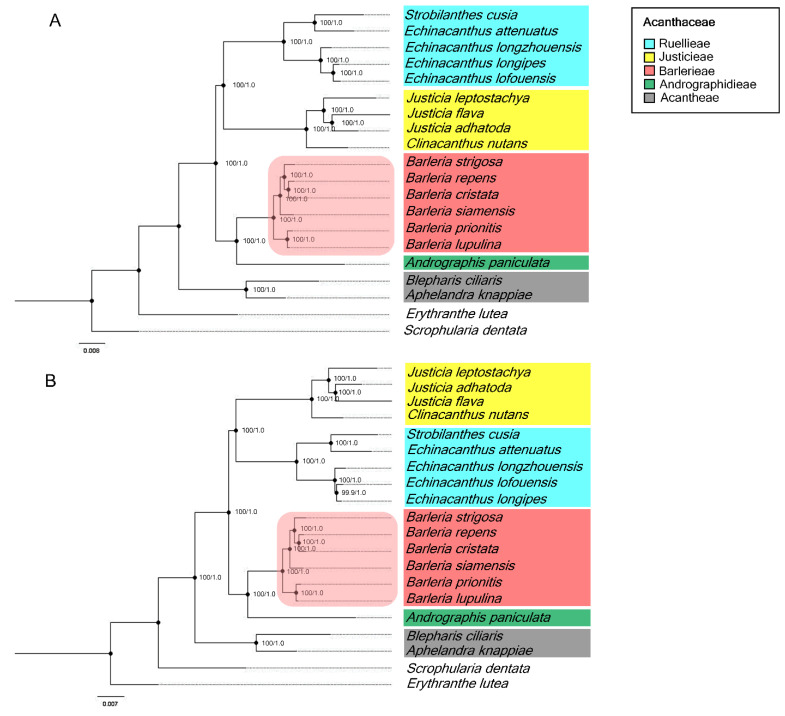
Phylogenetic relationships of the twenty chloroplast sequences inferred from the maximum likelihood (ML) and the Bayesian inference (BI). The numbers associated with each node represent bootstrap support value/posterior probabilities (PPs); (**A**) a phylogenetic tree from the complete chloroplast sequences; (**B**) a phylogenetic tree from the protein-coding sequences (CDSs); the colors were used to classify the Acanthaceae tribes. Two species, *Erythanthe lutea* (Phrymaceae) and *Scrophularia dentata* (Scrophulariaceae), were used for the outgroups.

A phylogenetic tree, constructed based on the complete chloroplast genomes and CDS sequences, revealed that all six species of *Barleria* were clustered under a single clade with the outer outgroup of *Scrophularia* and *Erythranthe* discriminating perfectly (Figure 6A,B). The phylogenetic relationship of *Barleria* was well-resolved. Additionally, the inner outgroups of different genera, including *Strobilanthes*, *Echinacanthus*, *Justicia*, *Andrographis*, *Blepharis*, and *Aphelandra*, were a separate complete group with a strong bootstrap support value and posterior probabilities (PPs). At the tribe level, Barlerieae are closely related to Andrographidieae (Figure 6A). Similarly, the CDS sequence of the *Barleria* species formed a monophyletic clade with the identical topology as the chloroplast genome dataset (Figure 6B). Identically, the BI trees performed a consensus on the ML trees with strong supporting values of posterior probabilities. 

**Figure 7:**
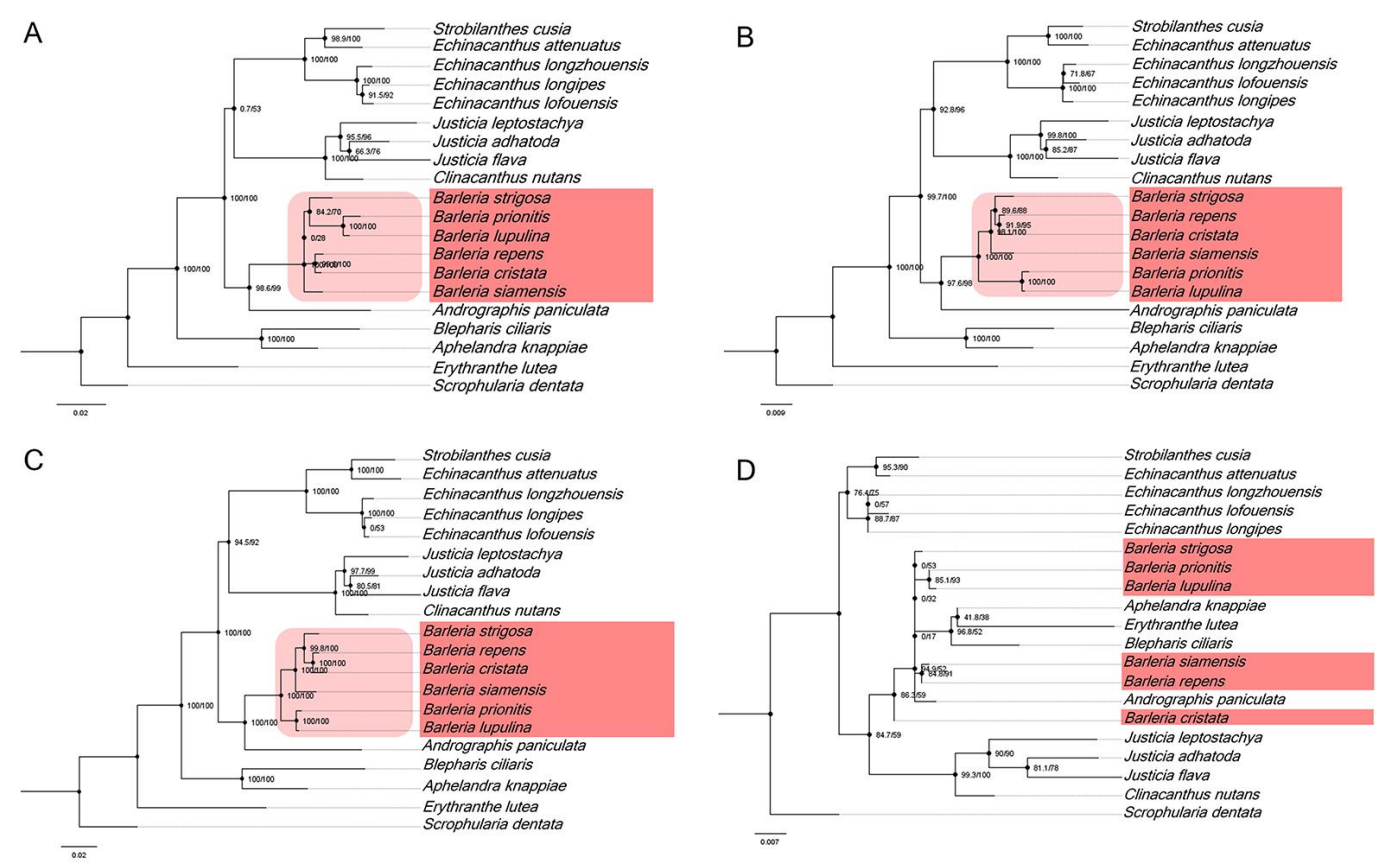
Phylogenetic relationships from four nucleotide hotspot regions, including (**A**) *ccs*A-*ndh*D, (**B**) *ndh*A-*ndh*H-*rps*15, (**C**) *ycf*1, and (**D**) *trn*N-GUU-*trn*R-ACG. The numbers associated with each node represent bootstrap support values.

In addition, the four divergent hotspot regions (Figure 5) that were identified in the Barleria cp genome have been used for a phylogenetic analysis. The results revealed that three of the four regions, including *ccs*A-*ndh*D, *ndh*A-*ndh*H-*rps*15, and *ycf*1, can be utilized for identification and are clustered as a monophyletic group in the five *Barleria* species with a strong bootstrap support value (Figure 7A–C). Meanwhile, *trn*N-GUU-*trn*R-ACG cannot be used for species identification (Figure 7D). Therefore, the three mentioned divergent hotspot regions could be applied as a molecular marker for the *Barleria* species.

## 4. Discussion

The analysis of the five *Barleria* cp genomes compared to *B. prionitis* was performed and was found to be 152,217 bp in length [23]. The identification of various *Barleria* species should be performed in order to use the correct species for the right purpose, corresponding to the bioactive compounds contained and their properties in natural product creation for humans, such as medicine, cosmetics, and nutraceutical supplements [1,3,4,5,6,7]. Several species of *Barleria* are known for their medicinal or ornamental values [6,43,55]. There have been studies reviewing the traditional, phytochemical, and pharmacological properties of certain species of *Barleria*, such as *B. lupulina* and *B. prionitis* [56,57,58]. Ghosh et al., 2012 [59] have studied the vegetative and floral characters of *B. cristata*, *B. lupulina*, *B. prionitis*, and *B. strigosa*. They reported that there is much variation in both characters, including that the leaves are more variable in *B. prionitis*, while *B. strigosa* and *B. cristata* have very variable features in their floral organs, and *B. cristata* has various color of flowers. However, species identification should not be too difficult, and it is performed by simply using a short nucleotide sequence of genes, three plastid intergenic spacers (*trn*S-G, *ndh*F-*rpl*32-*trn*L (UAG)), and the nuclear region nrITS. 

The sequences can create a monophyletic *Barleria* and provide support for the two currently recognized subgenera [2]. However, by systematics, getting large amounts of data, such as full plastid sequences, would be more accurate than extracting each of these mentioned short areas, which has the potential for errors. For example, Poeaim et al., 2013 [8] investigated five species and 16 samples of Thai *Barleria*, two *Rhinacanthus nasutus*, and *Andrographis pani-culata* species for the outgroup using the *trn*L-*trn*F sequences. The two sequences divided each species of *Barleria*, but it was noted that *A. paniculata* was inserted in the in-group of the *Barleria* species. Presently, no one has comprehensively reported on the species within *Barleria* using the existing literature in a clear and concise manner [7]. Therefore, the cp genome variations of each studied *Barleria* species have been used as an accurate marker for species authentication. 

The first difference between the five studied species is the bp length, including 151,977, 152,273, 152,324, 152,236, and 152,272 bp in *B. cristata*, *B. lupulina*, *B. repens*, *B. siamenesis*, and *B. strigosa*, respectively. Subsequent articles which need to be mentioned for their different points are the following: 53, 61, 55, 56, and 58 SSR contigs of *B. cristata*, *B. lupulina*, *B. repens*, *B. siamensis*, and *B. strigosa* in addition to various repeat types starting from mono-long repeats; identical gene content adjacent to the region borders except for *B. strigosa*, which revealed a shorter *ndh*F gene sequence and a lack of the *ycf*1 gene; slightly different genetic distance values of 0.00241–0.01125, which could be used for species identification; three distinct gaps of nucleotide variations among the species in the form of insertion–deletion located at the intergenic spacer regions of the LSC and CDS of the SSC; 2920, 1869, 806, and 121 variable sites in the LSC, SSC, and IR sequences; four divergent hotspot regions found in all *Barleria* species in the SSC region; nucleotide diversity values ranged from 0.0267 to 0.0365. Of these four regions, the three regions including *ccs*A-*ndh*D, *ndh*A-*ndh*H-*rps*15, and *ycf*1 can be employed for identification and authentication as a marker of the five studied *Barleria* species. Finally, the genetic relationships of the *Barleria* and the 13 genera, as shown in the family Acanthaceae, called the inner outgroup, and the different families Scrophularia (Scrophulariaceae) and Erythranthe (Phrymaceae) showed results in agreement derived from phylogenetic trees constructed from the cp genome and CDS sequences. The six species of *Barleria* under a single clade as a monophyletic group with a different inner group as genus and two species as an outer outgroup were perfectly discriminated. These results are useful for the identification and authentication of the studied *Barleria* species. Additionally, genomic knowledge confirms the potential of morphological characters.

## 5. Conclusions

Due to the advanced technology of genomic sequencing, the genetic data revealed a high output in terms of completeness and accuracy of the dataset for genetic relationships in a systematic study. For this research, the complete chloroplast genome sequences have been employed for a genetic study of five *Barleria* species. Although the cp genome of these species expressed similar features and organization, it contained slightly different characters in size (151,997–152,324 bp) and other features, including the following: pentanucleotide repeats detected in *B. lupulina* only; reverse long repeats identified in *B. lupulina* and *B.strigosa*; a shorter *ndh*F gene and a lack of the *ycf*1 gene at the JSB border of *B. strigosa*, as well as an insertion–deletion variation; three divergent hot spots regions used for the identification and molecular markers in the five *Barleria* species. The genetic datasets of the complete cp sequence and CDS can be generated for the *Barleria* species, clustering as a monophyletic group in the Barlerieae tribe, which is in line with the hypothesis. 

## Figures and Tables

**Table 1:** Chloroplast genome features of *Barleria* species.

Plant Species	Length (bp)				G-C Content (%)				Total of Genes	CDS	tRNA	rRNA	Collection Numbers	GenBank Accession Numbers
	LSC	SSC	IR	cpGenome	LSC	SSC	IR	cpGenome						
*B. cristata*	83,650	17,677	25,325	151,977	36.5	32.6	38.4	43.5	131	86	37	8	A. Chaveerach 1095	ON768801
*B. lupulina*	83,854	17,789	25,315	152,273	36.4	32.7	38.3	43.5	131	86	37	8	A. Chaveerach 1096	ON768802
*B. repens*	83,819	17,771	25,367	152,324	36.5	32.5	38.3	43.4	131	86	37	8	A. Chaveerach 1099	ON768803
*B. siamensis*	83,929	17,804	25,296	152,236	36.5	32.7	38.4	43.4	131	86	37	8	A. chaveerach 1098	ON768804
*B. strigosa*	83,804	17,715	25,315	152,272	36.5	32.4	38.3	43.5	131	86	37	8	A. chaveerach 1097	ON768805

**Table 2:** List of annotated genes in the five *Barleria* chloroplast genomes.

Category	Group of Function	Location	List of Genes
Self-replication related genes	Large subunit of ribosome proteins	LSC/IR/SSC	*rpl*2(x2) ^b^, *rpl*14, *rpl*16 ^b^, *rpl*20, *rpl*22, *rpl*23(x2), *rpl*32, *rpl*33, *rpl*36
	small subunit of ribosomal proteins	LSC/IR/SSC	*rps*2, *rps*3, *rps*4, *rps*7(x2), *rps*8, *rps*11, *rps*12 ^a^, *rps*14, *rps*15, *rps*16 ^b^, *rps*18, *rps*19
	DNA-dependent RNA polymerase	LSC	*rpo*A, *rpo*B, *rpo*C1 ^b^, *rpo*C2
	rRNA genes	IR	*rrn*4.5(x2), *rrn*5(x2), *rrn*16(x2), *rrn*23(x2) ^b^
	tRNA genes	LSC/IR/SSC	*trn*A-UGC(x2) ^b^, *trn*C-GCA, *trn*D-GUC, *trn*E-UUC, *trn*F-GAA, *trnf*M-CAU, *trn*G-GCC, *trn*H-GUG, *trn*I-CAU(x2), *trn*I-GAU(x2) ^b^, *trn*K-UUU ^b^, *trn*L-CAA(x2), *trn*L-UAA ^b^, *trn*L-UAG, *trn*M-CAU, *trn*N-GUU(x2), *trn*P-UGG, *trn*Q-UUG, *trn*R-ACG(x2), *trn*R-UCU, *trn*S-CGA ^b^, *trn*S-GCU, *trn*S-GGA, *trn*S-UGA, *trn*T-GGU, *trn*T-UGU, *trn*V-GAC(x2), *trn*V-UAC ^b^, *trn*W-CCA, *trn*Y-GUA
Photosynthesis related genes	Photosystem I	LSC/SSC	*psa*A, *psa*B, *psa*C, *psa*I, *psa*J
	Photosystem II	LSC	*psb*A, *psb*B, *psb*C, *psb*D, *psb*E, *psb*F, *psb*H, *psb*I, *psb*J, *psb*K, *psb*L, *psb*M, *psb*N, *psb*T, *psb*Z
	NADH oxidoreductase	LSC/IR/SSC	*ndh*A ^b^, *ndh*B(x2) ^b^, *ndh*C, *ndh*D, *ndh*E, *ndh*F, *ndh*G, *ndh*H, *ndh*I, *ndh*J, *ndh*K
	Cytochrome b6/f complex	LSC	*pet*A, *pet*B ^b^, *pet*D ^b^, *pet*G, *pet*L, *pet*N
	Cytochrome c synthesis	SSC	*ccs*A
	ATP synthase	LSC	*atp*A, *atp*B, *atp*E, *atp*F ^b^, *atp*H, *atp*I
	Rubisco	LSC	*rbc*L
Other genes	Maturase	LSC	*mat*K
	Protease	LSC	*clp*P ^c^
	Envelope membrane protein	LSC	*cem*A
	Subunit acetyl-CoA-carboxylase	LSC	*acc*D
	translational initiation factor 1	LSC	*inf*A
Unknown function genes	Conserved hypothetical chloroplast reading frames (ycf)	LSC/IR/SSC	*ycf*1, *ycf*2(x2), *ycf*3 ^c^, *ycf*4, *ycf*15(x2)

**Symbolic definition**: a = a gene that has been divided into two independent transcription units; b = a gene with a single intron; c = a gene with two introns; (x2) = duplicated genes located in the IR regions.

**Table 3:** Eighteen genes with introns in the five *Barleria* chloroplast genomes and length of the exons and introns.

List of Genes	Location	Size (bp)					Total Size (bp)
		Exon I	Intron I	Exon II	Intron II	Exon III	
*atp*F	LSC	446	698	143			1289
*clp*P	LSC	228	642	291	718	66	1948
*ndh*A	SSC	539	1071	551			2163
*ndh*B	IR	755	678	776			2211
*pet*B	LSC	5	735	641			1383
*pet*D	LSC	7	742	474			1225
*rpl*16	LSC	405	893	8			1308
*rpl*2	IR	392	671	434			1499
*rpo*C1	LSC	1621	797	430			2851
*rps*16	LSC	226	867	39			1134
*rrn*23	IR	198	2611				2810
*trn*A-UGC	IR	35	810	36			883
*trn*I-GAU	IR	41	939	34			1016
*trn*K-UUU	LSC	36	2452	36			2525
*trn*L-UAA	LSC	36	485	49			572
*trn*S-CGA	LSC	31	664	59			756
*trn*V-UAC	LSC	36	592	37			667
*ycf*3	LSC	154	727	226	695	127	1933

**Table 4:** Genetic distance analysis of the *Barleria* chloroplast genome.

	*B. strigosa*	*B. repens*	*B. cristata*	*B. siamensis*	*B. prionitis*	*B. lupulina*
*B. strigosa*	1.00					
*B. repens*	0.00574	1.00				
*B. cristata*	0.00601	0.00374	1.00			
*B. siamensis*	0.00813	0.00772	0.00792	1.00		
*B. prionitis*	0.01125	0.01097	0.01109	0.01109	1.00	
*B. lupulina*	0.01031	0.00990	0.01016	0.00998	0.00241	1.00

## Data Availability

The complete chloroplast genome sequences of the five *Barleria* species were submitted at NCBI (GenBank accession number: ON768801–ON768805).

## Abbreviations

cp	chloroplast genomes
LSC	a large single copy
SSC	a small single copy
IRs	a pair of inverted repeats
CDS	protein-coding sequence
SSR	simple sequence repeat
